# Managerial social capital in the context of transformation and its influence on individual performance, organizational growth, and psychological patterns: a study of literature review

**DOI:** 10.3389/fpsyg.2026.1798156

**Published:** 2026-05-11

**Authors:** Zhen Zhang, Yueyi Wu, Jiahui Gong

**Affiliations:** School of Finance, Jilin University of Finance and Economics, Changchun, China

**Keywords:** digital transformation, digital trust, employee performance, managers, organizational growth, social capital

## Abstract

Against the backdrop of accelerating digital transformation, managerial social capital serves as a critical strategic resource helping organizations adapt to digital environments and improve innovation through its formation, evolution, and operating mechanisms. This study combines CiteSpace bibliometric analysis with systematic review methods based on the PRISMA guidelines to delineate the theoretical origins, analytical frameworks, and research trajectories of managerial social capital across its structural, relational, and cognitive dimensions. Furthermore, the Mixed Methods Appraisal Tool (MMAT) is employed to ensure the rigor of the research process. It focuses on recent progress and theoretical contributions to employee performance, organizational growth, psychological patterns, and platform interactions in digital contexts. Existing research indicates that managerial social capital in digital contexts is shifting from structural embeddedness toward deeper cognitive and relational embeddedness. Accordingly, this paper explains how managers can use digital media to build affect-based trust and psychological safety, thereby encouraging employee innovation and strengthening psychological contracts. It also highlights the role of managerial social capital in bridging virtual and physical interactions and supporting tacit knowledge sharing. Future research should provide theoretical support and practical guidance for firms seeking to optimize managerial social capital, strengthen organizational resilience, and improve innovation performance during digital transformation by further examining how digital technologies affect individual rational behavior and trust mechanisms.

## 1 Introduction

Digital transformation has emerged as a key factor reshaping strategy and organizational change in the digital economy, with managers playing a critical role (Vial, 2019). Their social capital, defined as the relationships and networks they can mobilize to access information, support, and scarce resources, can shape organizational agility, coordination efficiency, and innovation performance (Nahapiet and Ghoshal, 1998; Adler and Kwon, 2002). Especially in the digital age, as the physical boundaries of businesses have dissolved, decentralization and remote work have become the norm, significantly weakening the traditional hierarchical control based on spatial proximity. In this context of heightened spatial alienation and increasingly fragmented communication, formal structural authority tends to become ineffective, and the social capital that managers build through digital platforms has consequently emerged as an irreplaceable “informal governance mechanism.” This mechanism not only accelerates the precise matching of innovation resources but also, through long-established norms of reciprocity and reputation-building mechanisms, stimulates the willingness of network participants to voluntarily share knowledge, thereby resolving the “free riding” problem inherent in open innovation.

As digital transformation deepens, managers’ social capital increasingly develops and operates through virtual communication and platform-mediated work, often discussed under the notion of e-leadership, changing how trust forms and is sustained. Prior research commonly links affect-based trust and cognitive trust to repeated face-to-face contact and long-term collaboration. While these forms of trust can still emerge in digital environments, the pathways might differ. Virtual interaction, digital reputation, and algorithmic systems shape how to evaluate credibility, contributing to distinct features of “digital trust” (Roman et al., 2019). The escalating significance of managerial social capital in the digital age lies in its capacity to project credibility, reshape employees’ psychological contracts, and facilitate cross functional cognitive synergy. In this context, digital technology acts merely as an amplifier that expands the reach and modalities of social capital. Without the foundation of trust embedded in social networks and the ability to mobilize resources, digital tools will merely serve as vehicles for information transmission and will be unable to translate into an organization’s capacity for ecosystem integration or competitive advantage. This is also the root reason why managers’ social capital enables companies in the digital age to break through innovation bottlenecks and achieve sustainable growth.

While digital technologies can expand access to resources and accelerate the flow of information, they can also weaken relational depth and jeopardize psychological contracts. In many digital work contexts, employees’ willingness to share knowledge and collaborate strongly depends on perceived trust in managers and colleagues, which is influenced by the design and use of digital tools, including transparency, the richness of communication channels, and the visibility of social cues (Spottswood and Wohn, 2020). Therefore, a micro-level focus on cognition and trust offers a useful lens for understanding how digital transformation affects everyday organizational behavior.

Although research on digital innovation has rapidly expanded, most social capital literature still centers on traditional, offline settings, whereas how digital technologies reshape the internal structure of managers’ social capital at the micro level remains understudied, including how these changes translate into individual and organizational outcomes (AlNuaimi et al., 2022). To address this gap, this paper reviews recent literature on managerial social capital in digital contexts, examining how managerial social capital influences employee performance, organizational behavior, and employee psychology through trust building and cognitive alignment. The aim is to clarify how these mechanisms evolve under digital transformation and provide guidance for organizations seeking to strengthen managerial social capital to support adaptability and innovation.

The subsequent sections of this paper are organized as follows: Section 2 elaborates on data collection, search protocols and research methodology. Section 3 traces the development and theoretical underpinnings of social capital in the management literature. Section 4 explores central research topics related to managerial social capital in the digital era. The final section concludes the paper, addresses its limitations, and suggests avenues for further investigation.

## 2 Data sources and research methods

### 2.1 Data sources

The data source for this study is the Web of Science Core Journal Citation Index (WoS), specifically the SCI and SSCI databases. This paper selects WoS as the primary database because it contains a more comprehensive and high-quality collection of academic papers in the social sciences, providing robust citation tracking essential for bibliometric analysis using CiteSpace.

Since there is no universally accepted definition of “managerial social capital” in the academic community—and given that it essentially represents a micro-level manifestation of social capital within organizational management contexts—we conducted an advanced search spanning the period from 2016 to 2026. To maximize both the coverage and accuracy of the literature search, this study developed a full-text thematic search strategy that combines core concepts with the research subject. The specific search query is: TS = “(social capital” OR “social relations” OR “social ties” OR “social network”) AND TS = “manager*.” This study adopts “manager” as the core keyword, as the term covers leadership and supervisory roles across different hierarchical levels in an organization. In contrast to more restrictive terms such as “CEO” or “executive,” using “manager” reduces selection bias in the search and avoids overlooking key participants—including middle managers and project managers—who are vital to the digital transformation process. This design helps improve the comprehensiveness of the retrieved literature and lowers the likelihood of omitting core studies. The initial search yielded 2,188 articles.

We subsequently screened the literature in strict adherence to the PRISMA guidelines. To ensure methodological rigor, we applied a set of pre-specified inclusion and exclusion criteria. Studies were eligible for inclusion if they: (1) were published in English-language peer-reviewed academic journals between 2016 and 2026; and (2) centered explicitly on managerial or leadership social capital. Articles were excluded if they: (1) took non-academic or non-peer-reviewed forms (e.g., conference proceedings, book chapters, editorials); (2) adopted a purely macro-level, societal perspective on social capital or framed it merely as a counterpart to private capital; or (3) lacked clear conceptual or empirical links to organizational behavior and digital transformation.

**Figure 1:**
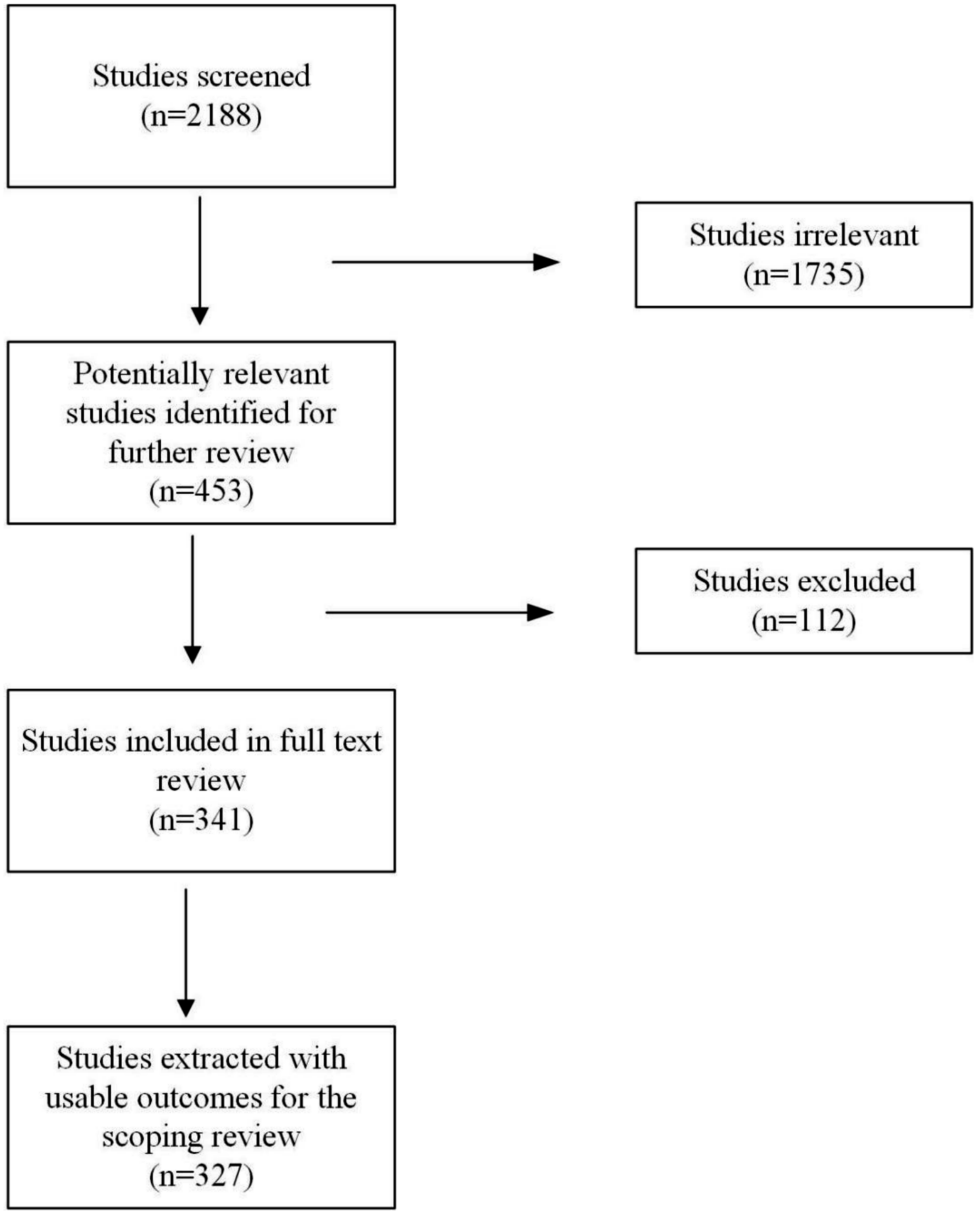
PRISMA diagram.

Initial title and abstract screening excluded 1735 clearly irrelevant articles, leaving 453 potentially relevant records for secondary screening. During this stage, we removed 112 non-academic publications, with the remaining 341 studies progressing to full-text evaluation. Following comprehensive full-text assessment, studies without reportable empirical or analytical findings were excluded. In total, 327 valid studies were ultimately identified and included in the final systematic review (Figure 1).

### 2.2 Research methods

This study combines bibliometric analysis with a systematic literature review. First, CiteSpace visualization software is used to conduct quantitative analyses of the selected literature. The study employs keyword clustering analysis, timeline mapping, and emergent term identification to systematically reveal the field’s developmental trajectory and overarching characteristics over the past 10 years. Building on this foundation, a literature analysis approach is employed to interpret and organize the content of relevant, frequently cited papers in related domains, including managerial social capital and digital transformation. Finally, the current progress, key themes, evolving trends, and future directions in research on managerial social capital are synthesized to provide theoretical grounds and directional guidance for subsequent studies.

### 2.3 Quality assessment

To ensure methodological rigor and reliability, we conducted a quality assessment of the included studies. Because the selected literature on managerial social capital encompasses quantitative, qualitative, and mixed-methods designs, we employed the 2018 version of the Mixed Methods Appraisal Tool (MMAT). The MMAT is explicitly designed for the appraisal stage of systematic reviews, enabling the concurrent evaluation of various study designs.

Following MMAT guidelines, two independent reviewers assessed the methodological quality of the core articles synthesized in Section 4. Initially, each article was screened using two preliminary questions to confirm research clarity and data adequacy. Subsequently, evaluations were conducted using specific methodological criteria corresponding to each research type (qualitative research, quantitative descriptive research, and mixed methods studies). Responses were recorded as “Yes,” “No,” or “Cannot tell.” Any discrepancies between the reviewers were resolved through discussion or by consulting a third researcher (Table 1) (Baym, 2015; Brubaker, 2020; Lakhani and Panetta, 2007; Marres, 2017; Muthuri et al., 2009; Ostrom and Ahn, 2003).

**Table 1:** Quality appraisal of included studies using the Mixed Methods Appraisal Tool (MMAT).

Code	Screening quest		1. Quantitative descriptive studies					2. Qualitative studies					3. Mixed methods studies					Score	Comments
	S1	S2	1.1	1.2	1.3	1.4	1.5	2.1	2.2	2.3	2.4	2.5	3.1	3.2	3.3	3.4	3.5		
4	Yes	Yes	Yes	Yes	Yes	Yes	Yes	-	-	-	-	-	-	-	-	-	-	100%	PLS-SEM model accurately fits survey data with adequate reporting of mediation assumptions.
8	Yes	Yes	Yes	Yes	Yes	Yes	Yes	-	-	-	-	-	-	-	-	-	-	100%	Empirical survey uses suitable tools to test sustainability strategies, ensuring reliability.
11	Yes	Yes	Yes	Yes	Yes	Yes	Yes	-	-	-	-	-	-	-	-	-	-	100%	Robust network analysis and statistical methods applied to a well-defined managerial sample.
17	Yes	Yes	Yes	Yes	Yes	Yes	Yes	-	-	-	-	-	-	-	-	-	-	100%	Survey-based analysis with clear quantitative design; adequate reporting of managerial coaching effects.
18	Yes	Yes	Yes	Yes	Yes	Cannot tell	Yes	-	-	-	-	-	-	-	-	-	-	80%	Robust network and panel regression methods applied to a well-defined organizational sample.
22	Yes	Yes	Yes	Yes	Yes	Yes	Yes	-	-	-	-	-	-	-	-	-	-	100%	Quasi-experimental intervention data analyzed using appropriate regression and validation methods.
25	Yes	Yes	Yes	Yes	Yes	Yes	Yes	-	-	-	-	-	-	-	-	-	-	100%	Survey-based analysis applied to a well-defined strategic alliance sample; adequate reporting of assumptions.
27	Yes	Yes	Yes	Yes	Yes	Yes	Yes	-	-	-	-	-	-	-	-	-	-	100%	Robust macro-statistical methods applied to a large-scale aggregate dataset for governance indicators.
33	Yes	Yes	Yes	Yes	Yes	Yes	Yes	-	-	-	-	-	-	-	-	-	-	100%	Survey-based analysis using structural equation modeling effectively tests entrepreneurial factors.
36	Yes	Yes	Yes	Yes	Yes	Yes	Yes	-	-	-	-	-	-	-	-	-	-	100%	Multilevel statistical analysis applied to a well-defined sample to effectively capture trickle-down dynamics.
45	Yes	Yes	Yes	Yes	Yes	Yes	Yes	-	-	-	-	-	-	-	-	-	-	100%	Mediation model accurately fits survey data with adequate reporting of statistical assumptions.
48	Yes	Yes	Yes	Yes	Yes	Yes	Yes	-	-	-	-	-	-	-	-	-	-	100%	Mediation model accurately fits team-level survey data with adequate statistical reporting.
51	Yes	Yes	Yes	Yes	Yes	Yes	Yes	-	-	-	-	-	-	-	-	-	-	100%	PLS-SEM model accurately fits high-tech SME survey data with robust validation methods.
54	Yes	Yes	Yes	Yes	Yes	Yes	Yes	-	-	-	-	-	-	-	-	-	-	100%	Empirical survey data analyzed using appropriate statistical methods to explore SME navigation strategies.
57	Yes	Yes	Yes	Yes	Yes	Yes	Yes	-	-	-	-	-	-	-	-	-	-	100%	PLS-SEM model accurately fits large-sample survey data to test complex chain-mediating effects.
58	Yes	Yes	Yes	Cannot tell	Yes	Yes	Yes	-	-	-	-	-	-	-	-	-	-	80%	Comparative quantitative design using validated indicators ensures reliability and representativeness.
59	Yes	Yes	Yes	Yes	Yes	Yes	Yes	-	-	-	-	-	-	-	-	-	-	100%	Survey-based analysis with clear sampling design; validated indicators ensure reliability of workforce ties.
60	Yes	Yes	Yes	Yes	Yes	Yes	Yes	-	-	-	-	-	-	-	-	-	-	100%	Robust statistical methods applied to test mediating mechanisms in SME digital platforms.
26	Yes	Yes	-	-	-	-	-	Yes	Yes	Yes	Yes	Yes	-	-	-	-	-	100%	The qualitative, case-based design effectively captures distributed innovation dynamics in organizations.
47	Yes	Yes	-	-	-	-	-	-	-	-	-	-	Yes	Yes	Yes	Cannot tell	Yes	80%	Exploratory assessment uses suitable mixed-method tools, though qualitative integration details are limited.

Code corresponds to the reference number. Screening questions: S1. Are there clear research questions? S2. Do the collected data allow one to address the research questions? 1. Quantitative descriptive studies: 1.1. Is the sampling strategy relevant to address the research question? 1.2. Is the sample representative of the target population? 1.3. Are the measurements appropriate? 1.4. Is the risk of nonresponse bias low? 1.5. Is the statistical analysis appropriate to answer the research question? 2. Qualitative studies: 2.1. Is the qualitative approach appropriate to answer the research question? 2.2. Are the qualitative data collection methods adequate to address the research question? 2.3. Are the findings adequately derived from the data? 2.4. Is the interpretation of the results sufficiently substantiated by data? 2.5. Is there coherence between qualitative data sources, collection, analysis and interpretation? 3. Mixed methods studies: 3.1. Is there an adequate rationale for using a mixed methods design to address the research question? 3.2. Are the different components of the study effectively integrated to answer the research question? 3.3. Are the outputs of the integration of qualitative and quantitative components adequately interpreted? 3.4. Are divergences and inconsistencies between the quantitative and qualitative results adequately addressed? 3.5. Do the different components of the study adhere to the quality criteria of each tradition of the methods involved?

Taken together, the quality assessment indicates that the included literature generally adheres to rigorous research designs and data collection procedures, with a low risk of bias. This provides solid support for the subsequent theoretical research and analytical work in this paper.

## 3 Literature review

### 3.1 Origins and development of managers’ social capital

The concept of social capital originated in sociology. Bourdieu (1986) focused on micro-level individuals, defining social capital as the collection of actual or potential resources individuals acquire through their institutionalized relational networks. Putnam (1995) identified its core elements as social networks, norms of reciprocity, and trust, which can promote collective action and cooperation while enhancing social efficiency. Coleman (1988) explored social capital from a productive perspective, examining how it fosters human capital development and maintains social order.

Management scholars have increasingly focused on social capital since the 1990s, re-examining and revising the traditional “*Homo economicus*” assumption. Granovetter (1985) proposed the theory of embeddedness, arguing that managers do not make economic decisions in a vacuum but instead rooted in real-world social networks. Building on this foundation, Nahapiet and Ghoshal’s (1998) research marks a significant shift in social capital theory from macro-level sociological analysis to micro-level organizational management applications, introducing the concept of social capital into organizational science, and emphasizing that managerial decisions and behaviors do not occur in isolation. They noted that reducing transaction costs and facilitating the flow of tacit knowledge can transform social capital into an enterprise’s organizational advantage. With the rise of the knowledge economy and globalization at the beginning of this century, research on managers’ social capital has increasingly focused on how managers build and maintain diverse social networks to acquire critical resources, drive organizational innovation, and navigate complex environmental changes, collectively advancing the deepening and expansion of social capital research. The resource-based view (Lockett et al., 2009) regards social capital as a crucial strategic tool for firms to acquire key resources, emphasizing its heterogeneity and the difficulty of imitating it as an intangible asset. Social exchange theory is grounded in the principle of reciprocity, positing that social capital emerges from long-term trust and mutual commitments (Zhou et al., 2024).

### 3.2 Conceptual definition of managers’ social capital

Managers’ social capital represents a significant extension of social capital theory in business administration, denoting the aggregate of tangible and latent resources that managers can mobilize and integrate through their social networks. Defined as a proprietary strategic resource grounded in trust, norms, and reciprocity, managers’ social capital improves organizational performance and contributes to managers’ personal career achievements by reducing transaction costs, facilitating knowledge transfer, and enhancing environmental adaptability. Managerial social capital is rooted in managers’ or organizations’ relational networks and comprises resources that can be mobilized to secure tangible or potential benefits. The bridging perspective focuses on how managers acquire critical external information and resources by establishing cross-boundary social connections, while the bonding perspective examines the cohesion formed within organizations based on trust, shared vision, and a sense of belonging, and its impact on collaborative efficiency.

**Figure 2:**
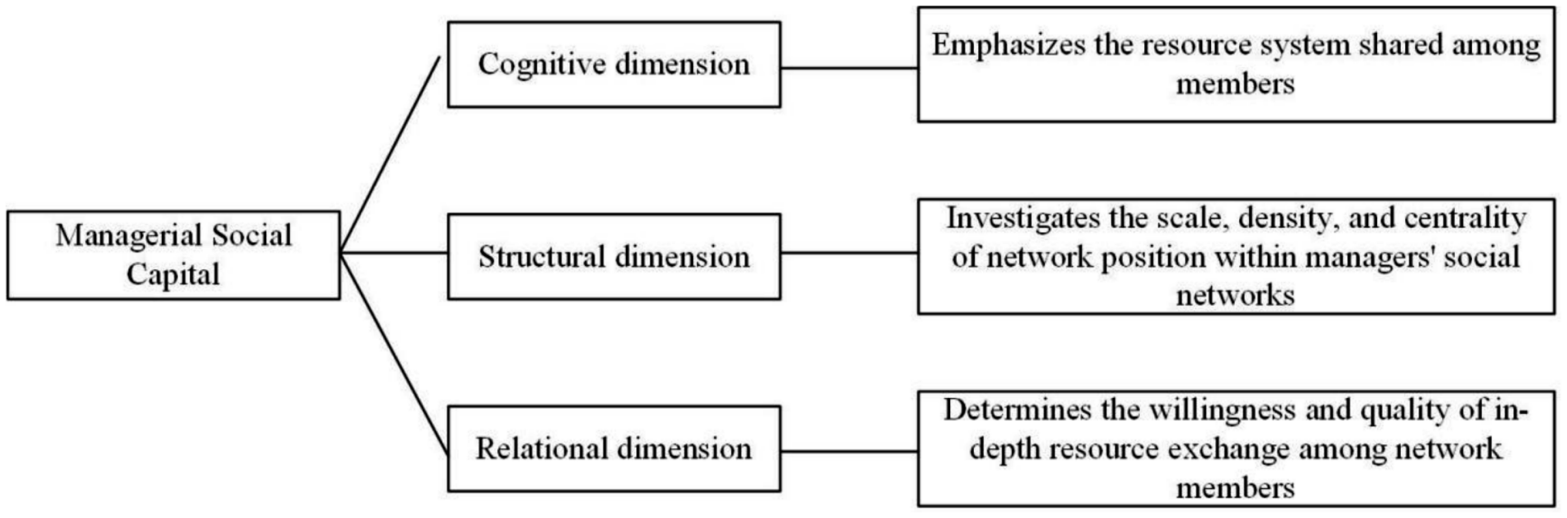
Three-dimensional analytical framework for managers’ social capital.

Figure 2 depicts Nahapiet and Ghoshal’s (1998) classic three-dimensional model, which provides a systematic analytical framework for social capital. The structural dimension focuses on topological characteristics, primarily examining the scale, density, and centrality position of managers’ social networks. The relational dimension emphasizes the asset attributes formed through long-term interactions, including trust, norms, obligations, and a sense of belonging. These factors directly influence the willingness and quality of deep resource exchanges among network members. The cognitive dimension emphasizes the shared resource systems among members, encompassing a common language, codes, narratives, and a strategic vision. Such high-level cognitive alignment ensures the accuracy of complex information transmission and serves as a critical prerequisite for organizational knowledge creation.

### 3.3 Evolutionary trajectory of managers’ social capital

**Figure 3:**
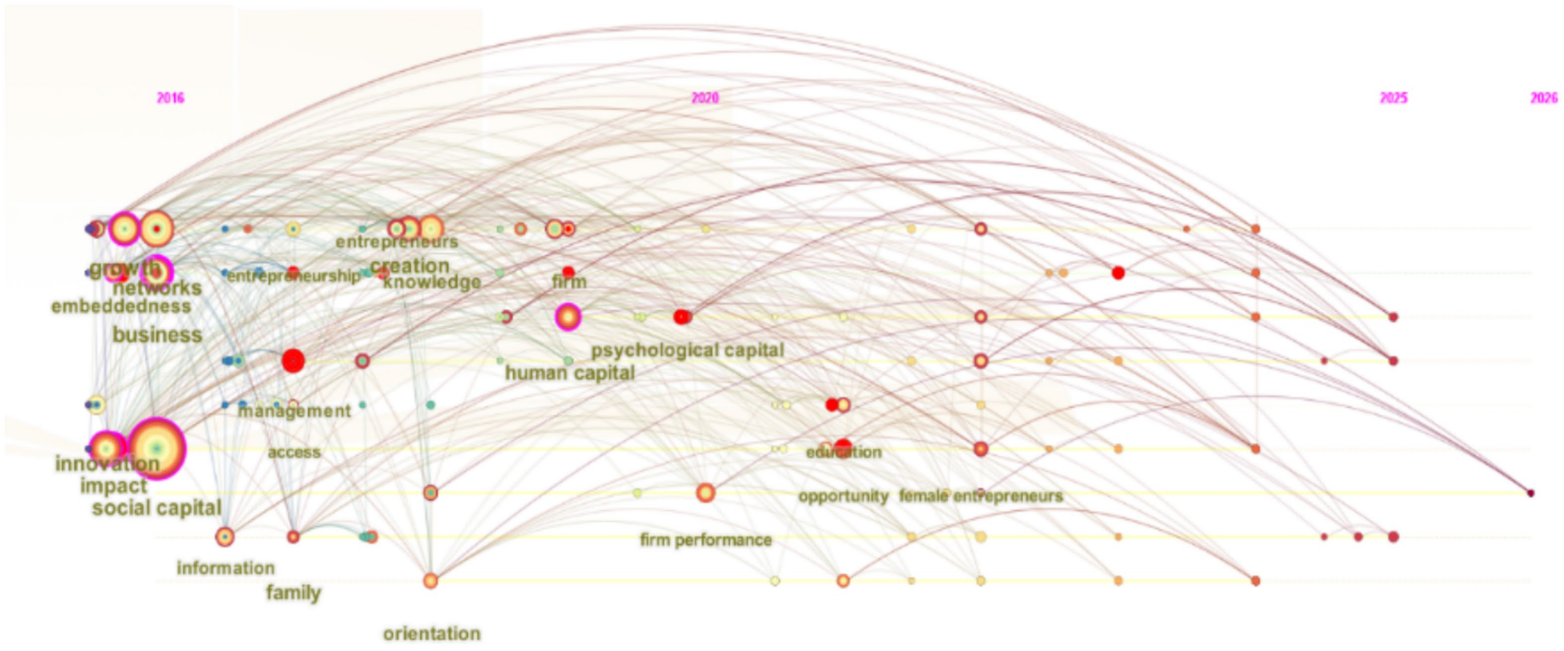
Evolutionary trajectory of research on managers’social capital.

**Figure 4:**
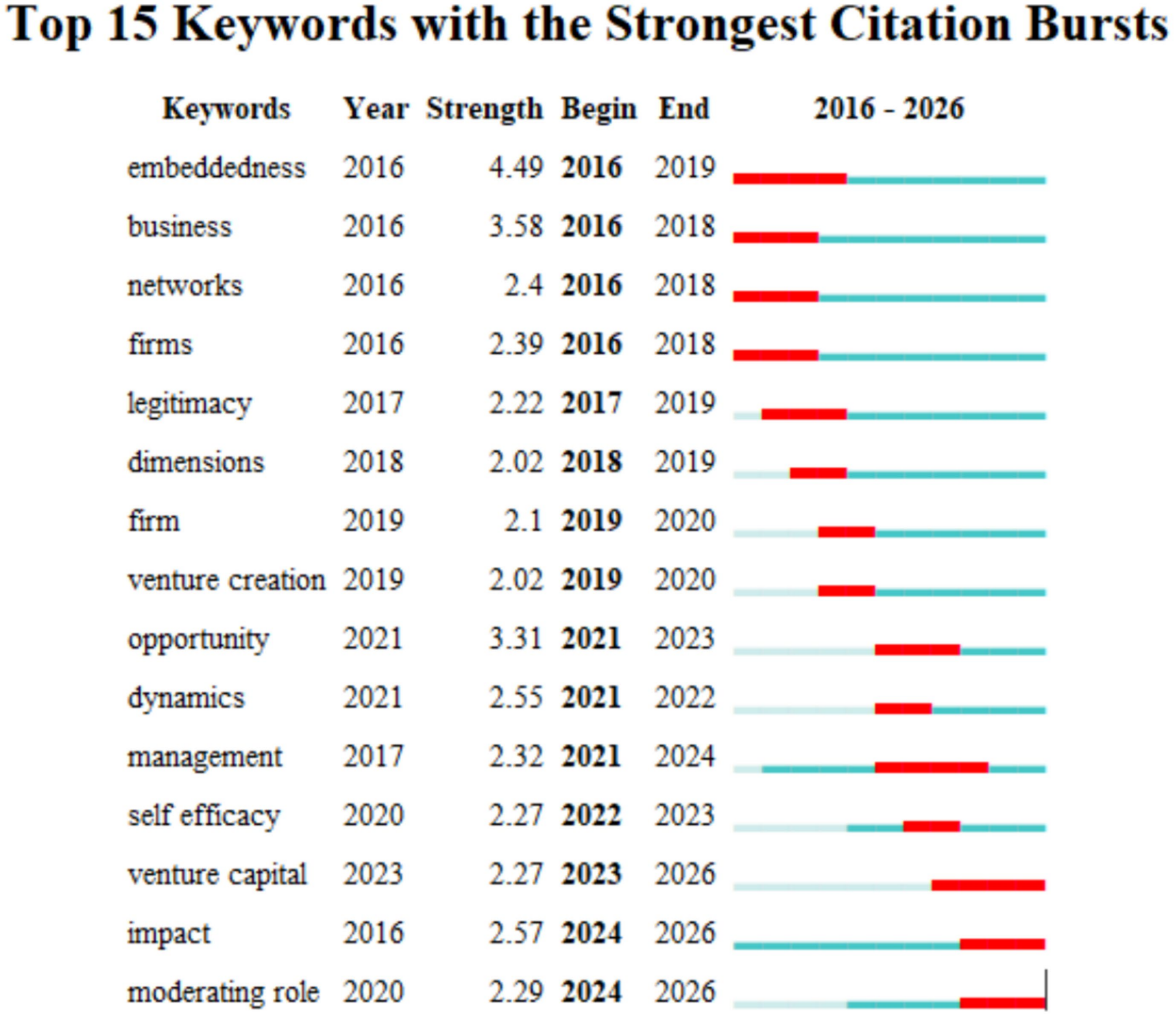
Research on managers’ social capital: keywords highlighted.

Scholars have increasingly focused on the contribution of managerial social capital to enterprise development. As revealed by the keyword timeline diagram (Figure 3) and keyword emergence test (Figure 4), early research (around 2016) centered on innovation, networks, business, and growth as core themes, representing foundational research directions. As the core keyword node, “embeddedness” maintains a high degree of salience. Research at this stage treats managers’ social capital as a static resource of network embeddedness, emphasizing the direct impact of its structural dimension on resource acquisition (McKeever et al., 2014). Around 2020, research topics expanded into areas such as human capital and psychological capital, marked by a significant increase in the prominence of nodes such as “management” and “enterprise.” This shift indicates that research has shifted from fundamental entrepreneurial elements toward individual entrepreneurial capital and enterprise entities, moving from macro-level factors to micro-level entity-level elements.

As the market environment continues to evolve, the prominence of keywords such as “regulatory role” and “risk capital” has gradually increased since 2023. Research during this phase has also shifted from the resource-based view toward the dynamic capabilities view (Kero and Bogale, 2023). The rise of digital technology has spurred research focused on virtual social capital, reflecting that managers can leverage networks to access tacit knowledge, thereby enhancing the breadth and depth of information search (Laursen and Salter, 2005). Furthermore, in their study on cross-cultural network building among multinational managers, Scherer and Palazzo (2010) noted that social capital possesses a politicizing function, enabling the coordination of institutional differences and the filling of regulatory vacuums through informal channels. This series of developments has gradually shifted the focus of managerial social capital research toward a dynamic capabilities perspective, placing greater emphasis on the internal mechanisms by which managers rapidly reconfigure their relational networks through streamlined, experiential processes in rapidly changing environments (Eisenhardt and Martin, 2000).

**Figure 5:**
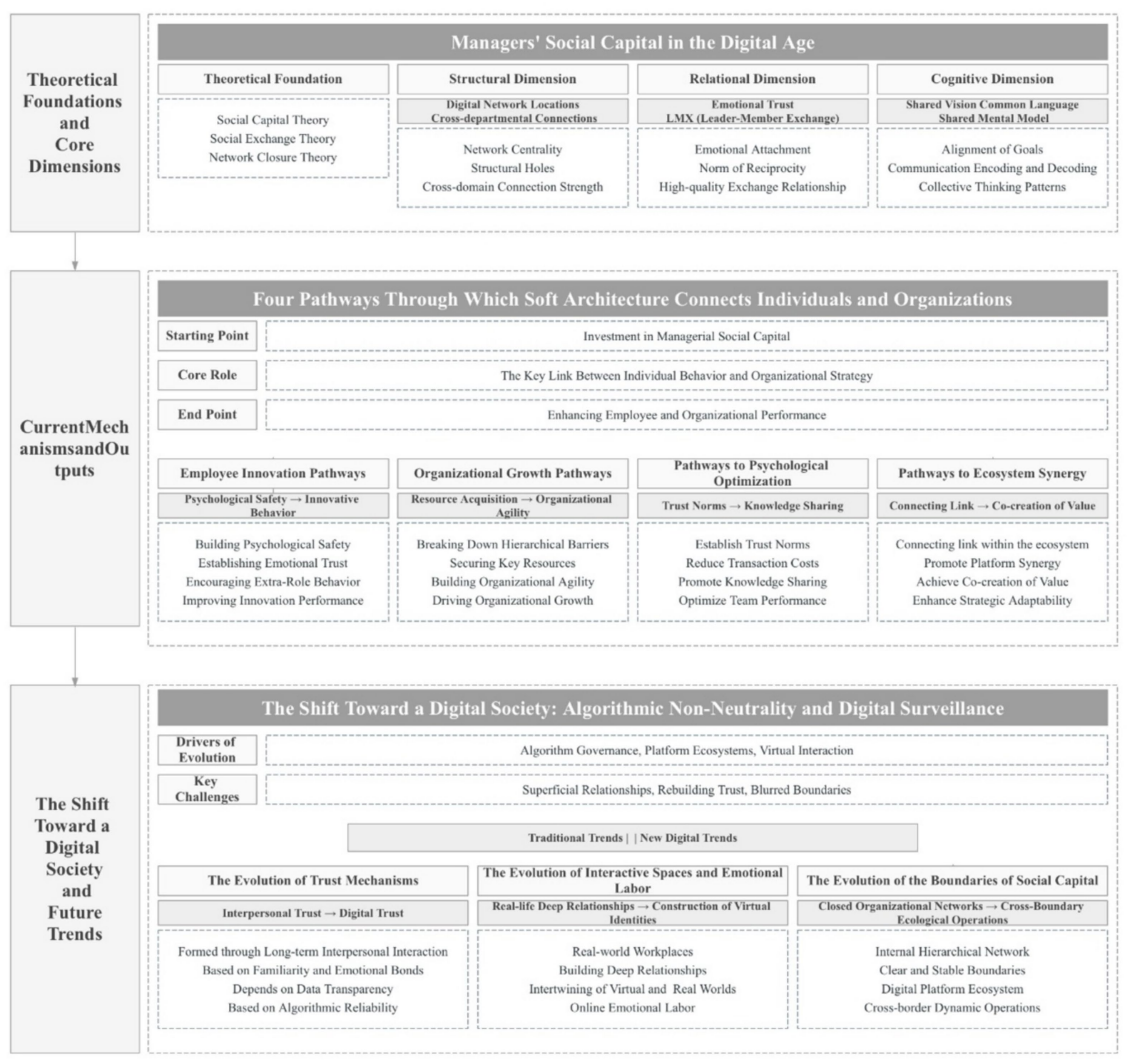
Trends in the evolution of managers’ social capital.

The theoretical origins and developmental trajectory of managerial social capital reveal that its various levels are both distinct and interconnected. Debates within this field originally stemmed primarily from differing research perspectives among scholars regarding related concepts. As the digital transformation continues to advance, research on managers’ social capital has gradually delved into the micro level, exhibiting a distinct trend toward digitalization. Therefore, this paper will focus on the digital context, systematically review research hotspots in this field, and summarize and project relevant issues. It aims to provide a reference for subsequent researchers to expand innovative studies on managers’ social capital and other strategic resources (Figure 5).

## 4 Prominent research areas

The evolution of organizational forms in the digital age has established managerial social capital as a cornerstone for driving digital transformation and enhancing organizational resilience. Current academic research primarily follows a logical chain of “micro-foundations—macro-outcomes—frontier contexts,” establishing a comprehensive research framework that spans from underlying mechanisms to the realization of value.

Specifically, at the micro-level, psychological patterns function as the internal catalysts through which social capital operates, determining the extent to which these social resources are integrated into individual behaviors. Employee performance is the direct result of social capital acting on individual employees, reflecting its actual impact on organizational members. From a macro perspective, organizational growth is the ultimate culmination of value delivery. As key platforms for optimizing the structure of social capital and building new networks of social relationships in the digital age, digital platforms provide a frontier research vehicle for this field. Accordingly, this paper will systematically review the current research trends and findings regarding managers’ social capital in the context of digital transformation, focusing on four dimensions: employee performance, organizational growth, employees’ psychological state and digital platforms.

### 4.1 Managerial social capital and employee performance

As the global economy gradually transitions from an industrial to a digital economy, the relationship between managerial social capital and employee performance has emerged as a core interdisciplinary concern (Wang and Cotton, 2017). The theoretical foundation of managerial social capital in organizational research primarily stems from social capital theory, which emphasizes individuals’ or organizations’ ability to acquire resources through social networks. Scholars typically measure social capital’s structural, relational, and cognitive dimensions (Kale et al., 2000; Kaufmann et al., 2009). Managerial social capital in the digital context represents an evolution of traditional social capital theory within digital media environments, denoting the relational networks managers construct through technological platforms, including resources, trust, and shared cognitive systems embodied (Spottswood and Wohn, 2020).

Existing research has identified structural and relational social capital as mechanisms through which managerial social capital influences employee performance. Structural social capital focuses on managers’ positions and connections within digital networks. For instance, cross-departmental networks established through digital collaboration tools can transcend traditional hierarchical constraints, enhancing employee innovation and advancing performance objectives by facilitating information sharing and collective action (Peethambaran and Naim, 2024; Qi et al., 2019). Cognitive social capital emphasizes the shared language, shared vision, trust, and norms formed within digital environments, which motivate employees to achieve common objectives (Chen et al., 2013). Consequently, social capital is widely recognized as a strategic resource that facilitates collaboration and resource integration within organizational performance (Fonti and Maoret, 2016).

Studies examining the relationship between managerial social capital and employee performance identify that social capital manifests through social networks, trust norms, and shared cognition, thereby influencing employee performance. Managerial social capital can empower human resource management practices, enhancing employee performance levels and strengthening employees’ dynamic capabilities (Wang et al., 2024). Nonetheless, the resulting differential allocation can trigger employees’ assessments of fairness and thus affect trust levels (Mawritz et al., 2012). At the theoretical level, the trust-building atmosphere that social networks foster reduces transaction costs and promotes knowledge sharing, thereby enhancing team performance (Chen et al., 2013). When employees are embedded within organizational networks formed by managers’ social capital, trust further strengthens their retention intentions and role-extending behaviors, indirectly driving performance improvement (Lee et al., 2014). Network closure theory indicates that tight internal connections enhance trust and information sharing, thereby boosting collective efficacy (Wang and Cotton, 2017). Social exchange theory also provides a framework for understanding performance enhancement, revealing the underlying mechanism by which employees repay managers for their resource provision with higher performance (Ahmad et al., 2023).

Empirical research has validated these pathways from both team and organizational perspectives. Managers can enhance employee performance by leveraging high-quality leader–member exchange (LMX) relationships built through their social capital, thereby providing greater resources and support (Heuvel et al., 2015). For instance, inclusive leadership strengthens LMX through social capital, effectively fostering employee innovation (Qi et al., 2019). In the digital context, managerial social capital further expands the boundaries of resource and information sharing via digital tools. For example, internal social media platforms improve work performance by enhancing relational capital, thereby promoting informal knowledge exchange (Lee et al., 2014). Simultaneously, enterprises successfully implementing digital transformation demonstrate significant improvements across performance metrics, including revenue growth, resource acquisition, and operational efficiency (Billi and Bernardo, 2025; Zhang et al., 2025).

The above research indicates that managerial social capital in a digital context positively influences employee performance and growth (Ellinger et al., 2010), significantly driving resource allocation, innovation promotion, and team collaboration (Wang et al., 2025). However, sufficiently refined theoretical models and cross-contextual empirical validation remain lacking regarding the scope and intensity of organizational-level digital social capital’s influence on performance. Accordingly, more rigorous and refined quantitative studies are necessary to enhance the explanatory power and persuasiveness of the relationship between digital managerial social capital and organizational performance.

### 4.2 Managerial social capital and organizational growth

Managerial social capital plays a pivotal role in organizational growth, primarily manifested in driving structural transformation, facilitating resource acquisition, and guiding cultural development.

Social capital guides organizations and helps them benefit from digital transformation. According to social capital theory, organizational growth depends on the relational networks in which it is embedded. Managers leverage external industry networks to learn, adopt, and combine best practices with the company’s own resources and development needs to optimize and adapt internal structures. Organizational agility is increasingly important in the digital context, with research indicating a bidirectional, synergistic evolutionary relationship between digitalization and organizational agility (Ciampi et al., 2022). Accordingly, SMEs can enhance competitiveness by improving organizational agility (Troise et al., 2022). Manager’s social capital plays a pivotal bridging role in this process by leveraging their network resources and integration capabilities to support the implementation of digital leadership, thereby fostering bidirectional synergy and mutual reinforcement between digital strategy and organizational agility (AlNuaimi et al., 2022).

The information transmission and collaborative integration functions of managerial social capital enable managers to occupy a central position within information exchange systems, ensuring their easier access to scarce resources and critical information. Managers can discern market demands and industry trends by connecting diverse network nodes such as suppliers, customers, and competitors (Burt, 1997). In the digital context, managers can leverage social capital to efficiently identify and integrate talent, technological tools, and data resources, thereby enhancing information processing efficiency and decision-making rigor (Yoo et al., 2010). Simultaneously, managers can leverage social capital through internal social platforms and industry-specific professional communities to rapidly assemble cross-functional teams to achieve precise resource-task matching. This process not only reduces information search costs but also drives internal organizational innovation by integrating heterogeneous knowledge (Lakhani and Panetta, 2007).

At the cultural level, managers cultivate social capital within the organization by shaping shared values and fostering a collaborative atmosphere. A positive organizational culture fosters trust and cooperation among employees, thereby supporting the effective implementation of organizational strategies (Ruggieri et al., 2023). Managers’ digital leadership plays a pivotal role in building employee trust, communicating core organizational values, and safeguarding external reputation, particularly in digital transformation contexts. This ability to shape culture enhances the organization’s adaptability and potential for sustained growth within dynamic environments.

### 4.3 Managerial social capital and employees’ psychological state

Managerial social capital significantly influences employee cognition and behavior through trust mechanisms, psychological safety, and psychological accessibility, thereby reducing psychological transaction costs within organizations. As the core element of social capital, trust originates from the “goodwill” embedded within relational networks (Adler and Kwon, 2002). Establishing tight relational networks with employees can help build affect-based trust, reducing employees’ defensive behaviors and suspicions of intent, thereby lowering the need for supervision and associated costs.

Social capital also enhances employees’ psychological safety and psychological accessibility, encouraging them to present a more authentic self and engage more fully in their work. Managers can leverage social capital to embed corporate social responsibility into daily organizational operations, thereby strengthening employees’ sense of work meaning while boosting their self-esteem and self-efficacy (Aguinis and Glavas, 2012). Research further indicates that positive interactions help employees integrate their “personal self” with their “organizational self,” enabling fuller self-expression (Bartel, 2001; Carmeli et al., 2007).

In the digital era, managers’ social capital has transcended traditional interpersonal networks, extending to virtual collaboration platforms and cross-departmental remote interaction networks. Roman et al. (2019) introduced the concept of “e-leadership” to address the challenge of building trust in non-face-to-face communication, emphasizing the use of ICT tools to convey trust and foster identification. For instance, managers leverage social media interactions and transparent information sharing to enhance employees’ trust in the organization and their identification with shared values.

### 4.4 Managers’ social capital and digital platforms

Digital technologies have transformed how enterprises create value, requiring managers to leverage digital platforms to foster the co-creation of enterprise value and drive corporate digital transformation. While traditional resource acquisition methods often rely on strong ties and localized networks, digital platforms emphasize the potential of weak ties and global networks. This paradigm reveals the dynamic value of managers’ social capital in digital environments, whose mechanisms transcend the limitations of traditional offline networks. Managers can access diverse information and resources through digital platforms, thereby breaking free from the constraints of information silos. Simultaneously, digital platforms enable managers to accumulate and utilize their social capital with greater scalability and replicability, rapidly replicating successful network operation models using platform tools and applying them to new markets or domains. Consequently, the accumulation and utilization of social capital becomes increasingly flexible and efficient.

The manifestations of managers’ social capital have transformed within digital platforms. In the structural dimension, network connectivity transcends geographical constraints through digital platforms, enabling managers to establish and maintain extensive business relationships more efficiently (Nambisan et al., 2017). In the relational dimension, digital reputation systems and algorithm-driven evaluation frameworks reinforce trust mechanisms within platform environments (Rysman, 2009). In the cognitive dimension, standardized platform interfaces and collaborative tools deepen shared language and values (Jacobides et al., 2018).

Managers can dynamically build and adjust social networks on digital platforms to respond to environmental changes, thereby enhancing corporate competitiveness. Managers’ social capital positively influences both business model innovation and the capacity enhancement of small and medium-sized enterprises (SMEs) through digital platforms (Xie et al., 2022). For resource-constrained SMEs, digital platforms can overcome traditional resource limitations and effectively enhance managerial capabilities (Li et al., 2017). Digital platforms have not only restructured how enterprises acquire resources but also redefined the operational logic of business ecosystems. Managerial capabilities and social capital synergistically influence corporate financing outcomes, thereby enhancing sustainable performance by improving supply chain finance efficiency and strengthening trust relationships within supply chains (Liu et al., 2025). In cultural and creative industries, the synergistic effect of digital literacy and managers’ social capital significantly enhances SMEs’ innovation performance.

Managerial social capital can effectively activate the value of platform relationships. For example, in highly connected platforms such as YouTube and the metaverse, managers of high-tech SMEs can leverage value realization through establishing norms of trust and collaboration enhance performance (Shahzad and Zhang, 2025). As the hub of the platform’s informal network, managers’ social capital serves as the key resource for connecting and activating network value, transforming the platform into a network node for acquiring critical resources. Meanwhile, managers actively leverage digital platforms’ sustainable infrastructure to accumulate and develop social capital through institutionalized design, continuously enhancing its quality. The complementary role division within platform ecosystems creates value-added opportunities for managers to consolidate and strengthen their social capital within interdependent roles through strategic coordination and resource integration (Jacobides et al., 2018). This mechanism is particularly evident in comprehensive platforms such as Alibaba, which not only provide a marketplace but also offer comprehensive support for cultivating and enhancing managers’ social capital through systematic designs such as credit systems, training services, and ecosystem partner programs (Min et al., 2018).

Existing studies indicate that platform type moderates the efficiency of social capital conversion: highly modular platforms rely more on managers’ structural social capital, while content-based platforms focus more on cognitive social capital (Sturgeon, 2019). However, this mechanism still faces challenges, including the digital divide (Jenkins et al., 2006), risks of platform monopolization, and the devaluation of social capital due to rapid technological iteration (Nambisan et al., 2017). Future research should focus on how to build more inclusive and efficient interaction mechanisms through platform design and the cultivation of social capital (Foss and Saebi, 2016).

## 5 Conclusions and research trends

### 5.1 Research findings

This study systematically traces the theoretical evolution and the prominent research on managerial social capital in the context of digital transformation. Through bibliometric and systematic literature analysis, it constructs a three-dimensional “structure–relationship–cognition” analytical framework. The study explores the operational mechanisms of this capital across four dimensions in digital contexts: employee performance, organizational growth, psychological patterns, and platform interactions. First, it stimulates employee innovation performance by fostering psychological safety and affect-based trust. Second, it drives organizational growth through resource acquisition and the development of agility. Third, it optimizes employee mental models by reducing psychological transaction costs. Fourth, it serves as a connecting link to promote ecological synergy and value co-creation between enterprises and digital platforms.

Managerial social capital plays an irreplaceable role as a “soft architecture” in corporate digital transformation, serving as a pivotal bridge between individual behavior and organizational strategy. By integrating an interdisciplinary perspective, this study analyzes the micro-mechanisms of managerial social capital during digital transformation. It provides a theoretical framework for subsequent scholars to explore how digital technologies reshape organizational trust systems, while also offering practical guidance for enterprises to enhance managerial digital literacy and boost innovation effectiveness through social capital management.

### 5.2 Research limitations

This study conducted a comprehensive systematic review and bibliometric analysis of managerial social capital in the digital context; however, the following limitations remain. First, regarding data collection, the literature search was limited to the Web of Science Core Collection. Although WoS is widely recognized for its coverage of high-quality academic publications, reliance on a single database may result in the omission of relevant studies indexed in other major databases such as Scopus and EBSCO, as well as important non-journal publications such as academic monographs or conference papers. Second, while the use of the specific keyword “manager” helps focus on the topic, it may exclude studies that employ alternative terms to describe similar micro-level relational phenomena, such as “leader networks” or “executive connections.” Finally, the bibliometric analysis in this paper primarily focuses on revealing macro-level research themes and trends, rather than quantitatively evaluating empirical relationships. Therefore, future research could incorporate meta-analytical methods to assess the specific effect sizes of managerial social capital on employee performance and psychological well-being across different digital contexts.

### 5.3 Future research directions

The rapid advancement of digital technologies is propelling research on managerial social capital into a more micro-level, dynamic, and contextualized phase. Overall, this field is experiencing a distinct “digital sociological turn.” Drawing on concepts such as “algorithmic non-neutrality” (Rogers, 2013) and “digital surveillance” (Bauman and Lyon, 2013), future inquiries should prioritize the following avenues:

First, the transition from interpersonal trust to “digital trust.” Traditional cognitive trust, rooted in long-term interpersonal interactions, is gradually shifting toward a “digital trust” predicated on data transparency and algorithmic reliability (Roman et al., 2019). Future studies must address how managers balance traditional interpersonal dynamics with algorithmic logic in human-machine collaborative environments, and how they cultivate new trust paradigms mediated by data.

Second, micro-interactions and emotional labor within intertwined physical-virtual networks. In the digital age, “connective relationships” (Licoppe, 2004) and “online communities” have created a new arena for capital, but they also pose potential risks such as the superficiality of relationships and the instrumentalization of interactions (Turkle, 2011). Future research could focus on managers’ “virtual identity construction” on digital platforms (Gálik, 2019), online self-presentation, and the resulting emotional labor, exploring how managers maintain deep relationships and achieve cognitive synergy amid fragmented digital interactions.

Third, the cross-boundary orchestration of social capital in digital ecosystems. As organizational boundaries become increasingly blurred, managers’ social capital is no longer confined to the closed confines of the organization or traditional hierarchical networks. The study should examine how managers, acting as “boundary spanners,” build cross-boundary networks and foster a sense of collective efficacy within complex digital platform ecosystems. At the same time, attention should be paid to the psychological impact of this process of cross-organizational network restructuring on managers, such as role conflicts arising from multiple networks, psychological empowerment, and the evolution of professional identity.

Fourth, the sustainable management of social capital under digital surveillance. While digitalization enhances connectivity, it also blurs the line between digital surveillance and privacy, potentially leading to “social overload” and the excessive instrumentalization of social capital by managers, which in turn can have a negative impact on employee mental health and the organizational atmosphere. To this end, conducting in-depth research into the harms caused by digitalization will help refine studies on the dual nature of social capital in a digital context. Specifically, managers should actively leverage the buffering role of their social capital to enhance employees’ psychological resilience and build digital trust, thereby fostering a people-centered, sustainable digital workplace ecosystem.

## Data Availability

Publicly available datasets were analyzed in this study. This data can be found at: https://webofscience.clarivate.cn/, http://webofscience-clarivate-cn-s.vpn1.jlufe.edu.cn:8118/wos/woscc/basic-search.
